# Narrowing the focus: a toolkit to systematically connect oncogenic signaling pathways with cancer phenotypes

**DOI:** 10.18632/genesandcancer.112

**Published:** 2016-07

**Authors:** Katherine R. Singleton, Kris C. Wood

**Affiliations:** ^1^ Department of Pharmacology and Cancer Biology, Duke University, Durham, NC, USA

**Keywords:** functional genomics, gain of function screens, drug resistance, cancer genomics, signaling pathways

## Abstract

Functional genomics approaches such as gain- and loss-of-function screening can efficiently reveal genes that control cancer cell growth, survival, signal transduction, and drug resistance, but distilling the results of large-scale screens into actionable therapeutic strategies is challenging given our incomplete understanding of the functions of many genes. Research over several decades, including the results of large-scale cancer sequencing projects, has made it clear that many oncogenic properties are controlled by a common set of core oncogenic signaling pathways. By directly screening this core set of pathways, rather than much larger numbers of individual genes, it may be possible to more directly and efficiently connect functional genomic screening results with therapeutic targets. Here, we describe the recent development of methods to directly screen oncogenic pathways in high-throughput. We summarize the results of studies that have used pathway-centric screening to map the pathways of resistance to targeted therapies in diverse cancer types, then conclude by expanding on potential future applications of this approach.

## INTRODUCTION

### Characterizing the genetic and functional landscape of cancer cells

The advent of high-throughput sequencing led to an explosion of data on the nature and number of genetic alterations present across a broad array of cancer types. Thanks to the efforts of the Cancer Genome Atlas, the International Cancer Genome Consortium, and other groups, researchers now have more knowledge than ever about the genetic events that trigger the transition from normal cell to cancer cell [1]. Through this body of work, we now know much more about the variety of mutation rates and patterns among different cancer types, the involvement of processes not originally thought to impact cancer, and even the existence of new cancer-relevant genes [2]. Many of these genetic discoveries have had profound impacts on the treatment of cancer patients. For example, the discovery of activating mutations in the epidermal growth factor receptor (EGFR) helped explain the success of EGFR inhibitors in a subset of lung cancer patients, in the process revolutionizing the screening and treatment of these patients [3–5]. Further, while some genetic insights have led to a decrease in reliance on older histopathology-based tumor classifications, other discoveries have served to deepen our understanding of the genetic bases of these classifications [6, 7].

However, still much remains to be discovered about cancer genomics. Beyond contemporary questions like how genetic heterogeneity impacts tumor biology, the importance of rarely mutated genes, and the function of non-coding regions of the genome, there is much that we simply do not understand about the workings of cancer-relevant mammalian genes. For instance, in a 70 gene expression signature of poor prognosis in breast tumors, the function of 20 genes is poorly understood [8]. Similar proportions of poorly understood genes can be found in lists of the most commonly altered genes in cancer, predictors of relapse on therapy, and others [9]. Additionally, apart from large scale sequencing efforts, hypothesis driven research has demonstrated the functional importance of many non-mutated genes such as those required for signaling downstream of oncogenes and tumor suppressors. The ability to identify these functionally important yet non-genetically altered genes could be crucial to the development of new therapies. Given that such genes are unlikely to be directly identified through sequencing alone, there is a clear role for complementary analysis techniques.

Toward this end, functional genomics methods enabling the specific and rapid manipulation of individual genes, first through cDNA expression, then siRNA- and shRNA-mediated RNA interference (RNAi), and more recently using CRISPR technology, have profoundly impacted the functional dissection of cellular processes, particularly those involved in cancer. As it had for whole genome sequencing, the emergence of affordable massively parallel sequencing allowed for the true rise of large-scale functional genomic screening, bypassing the throughput limitations of arrayed screens. Broadly, functional genomic screens can be categorized as either gain-of-function or loss-of-function based on their effects on target gene function. As loss-of-function screens echo the action of the majority of pharmacological agents available for cancer therapy, these screens have received the most attention from researchers. Targeted or whole genome libraries of siRNA, shRNA, or CRISPR constructs can be utilized to assess the impacts of genetic loss on biological processes of interest. For example, numerous published screens have identified genes whose loss impairs tumor progression, cell cycle control, growth factor signaling, and migration and invasion [10–17]. Inversely, gain-of-function screens can be effectively used to screen the effects of ectopic or endogenous gene expression on phenotypes of interest. Although assembly and physiological expression of gain-of-function ORF libraries remains challenging, these approaches have successfully identified, for example, kinases whose activation can maintain phosphoinositide-3-kinase (PI3K) signaling [18] and proteins whose overexpression confers sensitivity or resistance to cytotoxic and targeted cancer therapies [19–26]. More recently, new screening methods making use of CRISPR-based gene activation have made it possible to perform gain-of-function screens from endogenous promoters without ectopic cDNA expression [27–29].

Finally, within the space of functional genomic screening, a particularly important subset of work has borrowed concepts from yeast genetics to search for interactions between either genes or chemical compounds. Termed ‘synthetic lethal’ screens, these approaches involve the introduction of libraries of genetic reagents into a cell population bearing a gene alteration or treated with a compound, where the effect of each genetic perturbation is compared to a matched control population to identify genes whose alteration provides a specific outcome, such as cell death, only in the context of the studied gene alteration or chemical compound. These screens have provided important information on potential combination therapies with the potential to improve initial therapeutic responses or suppress resistance. Many groups have identified potential targets to enhance the cytotoxic effects of cancer therapeutics such as paclitaxel in lung cancer, PARP inhibitors in breast cancer, HDAC inhibitors in osteosarcoma, imatinib in CML, and BRAF inhibitors in colon cancer and melanoma among others [30–37].

## APPROACH

### High-throughput mapping of the signaling pathways driving resistance

With respect to the development of targeted cancer therapies, the results of the past decade of cancer genomics raise important questions. How can gene products such as RAS family members and other proteins lacking pharmacologically addressable active sites be targeted? Perhaps more broadly, given that a diverse array of genomic alterations can initiate tumorigenesis, cause drug resistance, spark metastasis, or provide escape from immune surveillance, combined with the established capacity of tumors to evolve resistance to drugs targeting individual alterations, how will it be possible to design robust therapies?

Our growing understanding of cancer cell signaling networks provides a potential answer to these thorny problems. While diverse genomic alterations can drive phenotypic outcomes such as drug resistance, the net result of each alteration is often the redundant modulation of a single signaling pathway. In just one example, the wide array of resistance mechanisms in *BRAF*-mutant melanoma to RAF inhibitors (RAFi) include mutations in *NRAS, MEK* and *ERK* and the amplification and alternative splicing of *BRAF* [20, 38–41]. However, as diverse as these resistance mechanisms may seem, they all result in the reactivation of mitogen-activated protein kinase (MAPK) signaling in the presence of the original RAF inhibitor. This seems to suggest a hardwired predilection for MAPK signaling itself, regardless of the specific modification instrumental to its stimulation. Other resistance mechanisms to RAFi have been identified in melanoma such as alterations in *IGF-R, PIK3CA, PTEN* and *AKT* [42–44]. In all of these cases, the alteration is likely to drive resistance through activation of the PI3K pathway, an alternative signaling pathway capable of rescuing growth and survival in the context of MAPK pathway inhibition. Outside the setting of melanoma, most of the identified resistance mechanisms to targeted therapy involve either bypass and reactivation of the original driver pathway or activation of a similar pathway. The ability to cut through the overabundance of specific alterations capable of activating canonical growth, survival, differentiation and apoptosis pathways, and instead focus solely on the specific pathways themselves, may provide much needed simplicity to the field of drug resistance and the broader pursuits of cancer biology research.

With this goal in mind, our group set out to devise a method of systematically interrogating signaling pathways for their impact on oncogenic properties, with a specific focus on drug resistance. We first assembled a set of 17 signaling pathways that had been previously found to be frequent players in oncogenic processes (Table 1). This list was composed of the MAPK and PI3K pathways as introduced above as well as major pathways contributing to proliferation (JAK-STAT, estrogen receptor (ER), androgen receptor (AR), TGF-β, ERK5, Ral), survival (p53, BCL-2 family members, p38, Hippo), differentiation (Wnt, Hedgehog, Notch), and inflammation (JNK, NF-κB), with many of these pathways also having impacts on multiple phenotypes [45]. For each of these pathways, we next selected 1-3 well validated methods of either activating (oncogenic pathways) or deactivating (tumor suppressive pathways) each signaling pathway. For instance, in the case of PI3K signaling, a total of three activating constructs were selected. These include myristoylated-PIK3CA and -AKT1, which localize constitutively at the cell membrane to initiate downstream signaling, and the Q64L mutant of *RHEB* which locks the GTPase in its active, GTP-bound state, facilitating activation of mTORC1. All of the activating and deactivating strategies are summarized in Table 1. We then barcoded and cloned these constructs into lentiviral vectors in which transgene expression is driven by the human phosphoglycerate kinase 1 (PGK) promoter and selection can be achieved using the puromycin resistance gene.

**Table 1:** cDNAs activating defined oncogenic signaling pathways

Signaling pathway	Protein	Activating strategy	Validation method
Ras-MAPK	KRAS	G12V mutation	Western (P-ERK)
	HRAS	G12V mutation	Western (P-ERK)
	MEK1	S218D, S222D mutations	Western (P-ERK)
PI3K-AKT-mTOR	PIK3CA	myr-FLAG tag	Western (P-AKT)
	AKT1	myr-FLAG tag	Western (P-AKT, P-S6K1)
	Rheb	Q64L mutation	Western (P-S6K1)
NF-κB	IKKα	S176E, S180E mutations	Reporter (NF-κB_Luc)
	IKKβ	S177E, S181E mutations	Reporter (NF-κB_Luc)
Jak/Stat	JAK2	V617F mutation	Reporter (Stat_Luc)
	Stat3	A662C, N664C, V667L mutations	Reporter (Stat_Luc)
Wnt/b-catenin	β-catenin	S33A, S37A, T41A, S45A mutations	Reporter (TCF-LEF_Luc)
	GSK3β	K85A mutation	Reporter (TCF-LEF_Luc)
	β-catenin	S33Y mutation	Reporter (TCF-LEF_Luc)
JNK	JNK2	WT overexpression	Reporter (AP1_Luc)
	JNK2	Mkk7 fusion	Reporter (AP1_Luc)
ERK5	MEK5	S311D, T315D mutations	Western (ERK5 laddering)
	MEK5	myr-FLAG tag	Western (ERK5 laddering)
Notch	Notch1	intracellular domain only	Reporter (HES1_Luc)
	Notch3	intracellular domain only	Reporter (HES1_Luc)
p38	p38 (MAPK14)	WT overexpression	Western (P-p38)
	MKK6	S207E, T211E mutations	Western (P-p38)
Hedgehog	Gli2	truncation	Reporter (Gli_Luc)
	SmoM2	W535L mutation	Reporter (Gli_Luc)
TGFβ	TGFβR1	WT overexpression	Immunofluorescence (P-Smad2/3)
Mitochondrial apoptosis (intrisic pathway)	BCL2	WT overexpression	Western (cleaved caspase 9)
	BCL-XL	WT overexpression	Western (cleaved caspase 9)
Death receptor apoptosis (extrisic pathway)	Caspase-8	C360A mutation	Western (cleaved caspase 8)
All apoptosis	Caspase-3	C163A mutation	Western (cleaved caspase 3/7)
Estrogen receptor	Erα	Y537S mutation	Reporter (ERE_Luc)
Androgen receptor	AR	V7 variant	Western (ARE_Luc)
Hippo	YAP2	FLAG-YAP2 (5SA)	Immunofluorescence (nuclear YAP)
	Lats2	kinase dead (K697R)	Immunofluorescence (nuclear YAP)
p53	p53	dominant negative R175H mutant	Reporter (p53_Luc)
Ral	Hras	G12V, E37G mutations	
	Rgl2	Rgl2-CAAX	
	RalA	G23V (two forms - full and mature peptide)	

**Figure 1:**
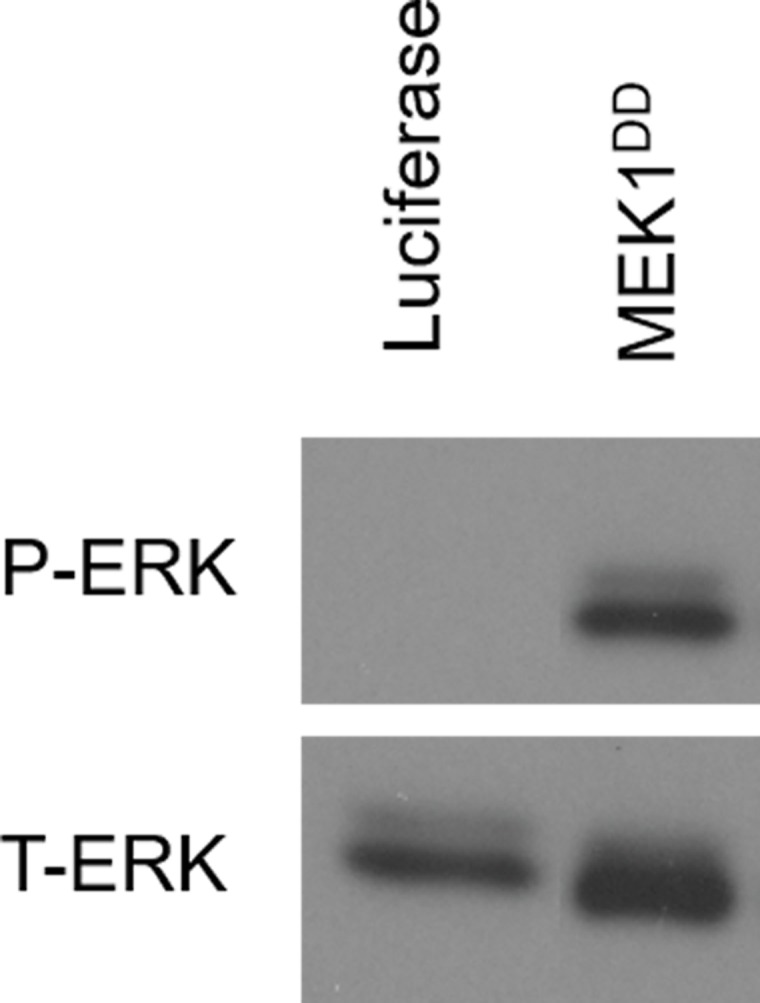
Example of pathway activating construct activity validation Target cells (shown here, 293T) were infected with constructs encoding individual pathway activating constructs. Following selection with puromycin, whole cell lysates were collected and immunoblotted for the appropriate markers of pathway activation. For the MAPK pathway activator, constitutively active mutant of MEK1, phospho-ERK was used as an indicator of proper pathway activation.

**Figure 2:**
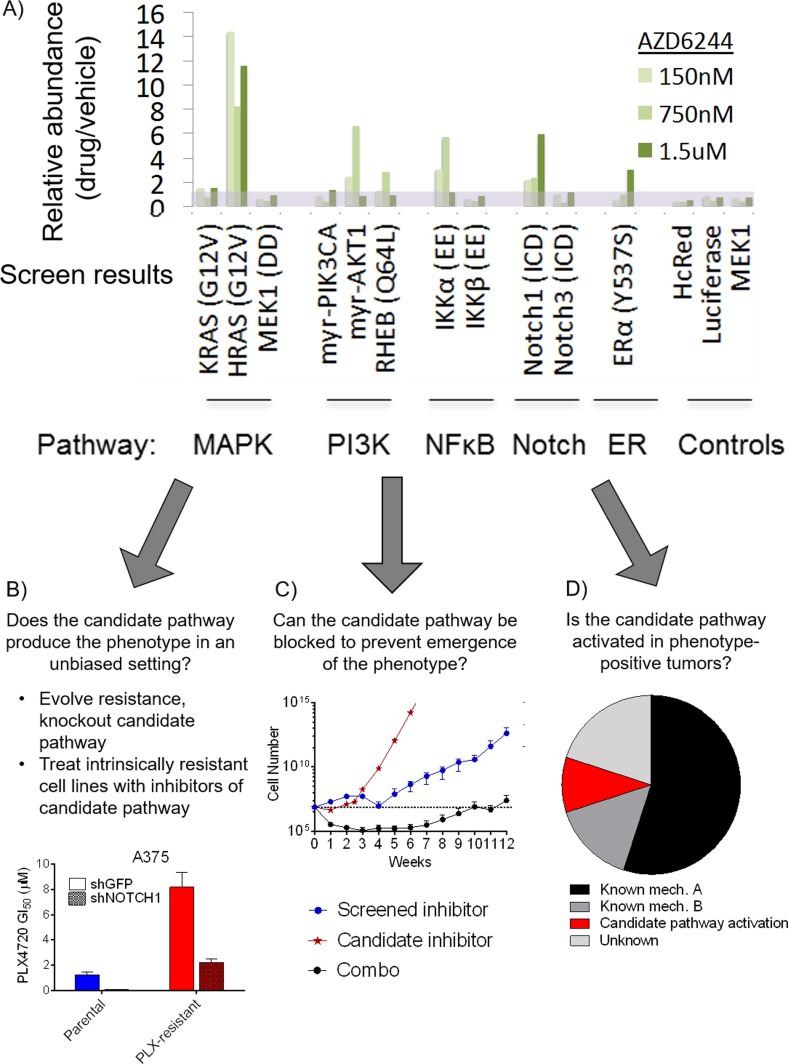
Pathway activating screen results and example validation methods **A.** Results of a pathway activating screen on UACC-62 cells for signaling pathways that provide resistance to the MEK inhibitor, AZD6244, at several doses. Abundance of scoring (MAPK, PI3K, NFκB, Notch1 and ER) and control (HcRed, luciferase, MEK1) pathway activators in drug-treated cells relative to diluent- treated cells is shown. **B.** Screen results can be used to guide investigation of naturally occurring mechanisms of the phenotype of interest. In the setting of resistance, evolved or intrinsically resistant cell lines can have the screen hits inhibited genetically or pharmacologically to investigate whether this reverses resistance. In this example, knock down of Notch1 sensitized MAPKi-resistant cells to the inhibitor. **C.** The screen hit can be blocked to determine if this prevents the emergence of the phenotype of interest. Here, cells were treated with the screen inhibitor, and inhibitor of the candidate pathway and the combo. Cells were counted weekly and it was observed that only the combination of inhibitors significantly delayed the emergence of resistance. **D.** Screen results can direct examination of tumor samples. In a survey of relapsed tumor biopsies, a most show evidence of known resistance mechanisms but a subset show activity, mutually exclusive with known mechanisms, of the candidate pathway.

In all, our library was composed of 36 constructs capable of modulating 17 major signaling pathways. All constructs were fully sequenced to confirm fidelity to the original source and 86% (31/36) of constructs were functionally validated by immunoblotting, reporter assay, or immunofluorescence to confirm proper engagement of each signaling pathway (Figure 1 and Table 1). These constructs can be used in arrayed or pooled screening formats. To investigate the utility of this library in the context of drug resistance, we first examined the setting of *BRAF-*mutant melanoma, one in which acquired resistance to RAF and MEK inhibitors has been well studied. Using a positive selection, pooled screening approach, we infected the *BRAF*-mutant melanoma cell line UACC-62 with the pooled library and treated populations of the cells with either vehicle control or the MEK1/2 inhibitor AZD6244 at multiple doses yielding between 20% to 80% growth inhibition (150 nM, 750 nM, and 1.5 μM). After 2-3 weeks of treatment, genomic DNA was isolated from each population and the construct barcode sequences were amplified and quantified by Illumina deep sequencing. By isolating pathway constructs that were over-represented in the drug treated populations *versus* the control populations, we could identify pathways that conferred a survival advantage to the cells expressing them under the selective pressure of MEK inhibition. This screen identified 5 pathways capable of conferring resistance (Figure 2). Three of these pathways, RAS-MAPK, PI3K and NF-κB, had previously been implicated as resistance mechanisms to RAF and MEK inhibitors, validating the ability of the method to identify biologically relevant resistance pathways. In addition, we found two novel pathways that were capable of conferring resistance, the Notch1 and ERα pathways. On the basis of the strength of this finding, we followed the preliminary screen with additional screens of 15 targeted therapies in relevant oncogene-driven cancer cell line models at multiple doses of each drug. While indicating novel resistance mechanisms in melanoma, breast cancer, colorectal cancer, myelofibrosis, and acute myeloid leukemia [46–49], overall, the screens provided several important observations. First, among the pathways screened, a few, such as RAS-MAPK, PI3K and Notch1, are capable of driving resistance to a broad number of inhibitors and in many cancer types. In contrast, other pathways were only observed to confer resistance in a few contexts. For example, NF-κB pathway activation could confer resistance to MAPKi in *BRAF*-mutant melanoma, but did not score as a resistance pathway in many other cancer types. Next, our evidence suggests that the further down a linear pathway one inhibits (e.g., inhibiting ERK *vs*. RAF), the more likely it is that the spectrum of resistance mechanisms will be dominated by alternative signaling pathways, while reactivation of the inhibited pathway is often the dominant mechanism of resistance to inhibitors higher up on the pathway. Put differently, in a competitive, pooled screen format, we find that resistance driven by simple pathway reactivation is favored over alternative survival pathways when possible, and that blocking driver pathways at lower nodes tends to favor the emergence of alternative resistance pathways. Finally, our screen provided some hope for the problem of managing resistance because, in general, fewer than 5 pathways was typically capable of providing resistance in a given context.

While offering an intriguing global picture of the oncogenic pathways that are capable of regulating a particular phenotype, it is essential to recognize that the hits uncovered in a pathway activation screen, like other gain-of-function screens, are merely candidates until further confirmatory validation experiments are performed. The screen is capable of guiding researchers to targets with a good probability of physiological and clinical relevancy, however, the limitations of the method necessitate a robust and thorough confirmatory workflow. We have found that the validation of the screens is best approached using a combination of methods in three general categories (Figure 2). First, using the example of drug resistance, cells can be interrogated for a natural propensity of the nominated pathway to be selected for in an unbiased setting. For example, cell lines with either evolved or intrinsic resistance can be assessed for the capacity of inhibitors of the nominated pathway to reverse resistance. We validated the finding that Notch1 activation can drive resistance to MAPK pathway inhibitors in *BRAF* mutant melanomas by evolving resistance to RAF (PLX4720), MEK (AZD6244) and ERK (VX-11E) inhibitors in 6 *BRAF* mutant melanoma cell lines, ultimately developing 18 pooled and 84 clonal resistant derivatives. Among these, 39% of the pooled and 29% of the clonal populations could be resensitized to MAPK inhibition through shRNA mediated knockdown of Notch1. Similarly, we validated the finding that MCL-1 and BCL-X_L_ up-regulation can drive resistance to the potential to be used to address many other questions in selective BCL-2 inhibitor ABT-199 (venetoclax) in acute myeloid leukemia (AML) by demonstrating the resensitization of cells with acquired resistance through knockdown or small molecule inhibition of these candidate resistance proteins [48]. Finally, we validated Ras effector pathways PI3K and MAPK as drivers of acquired resistance to JAK inhibitors in *JAK2* mutant cells using a similar approach in cells with acquired JAKi resistance [47]. A second validation approach is to demonstrate that by blocking the pathway in question, evolution of the phenotype it drives is either delayed or prevented entirely. For example, using this approach, we have substantiated screening hits by showing, for example, cancer biology. For example, Weinberg and Hanahan have famously implicated a defined set of cellular and tissue functions as the ‘hallmarks of cancer” [52]. Some of these hallmarks remain poorly understood from a signaling pathway and drug targeting perspective. It is straightforward therefore to envision the use of pathway activating screens to determine the relative impact of various pathways on hallmark phenotypes in diverse cancer models. Additionally, while library-based screening methods have thus far been mostly limited to *in vitro* cancer cell line applications, it is exciting to consider the additional information that could be gleaned from an *in vivo* application of these approaches [53–55]. Expanded that inhibition of BCL-X_L_ and MCL-1 can prevent the pathway focused screens could shed light on enduring development of resistance to BCL-2 inhibition in AML [48], and inhibition of the MAPK pathway with MEK inhibitors can prevent the development of resistance to EGFR inhibitors in colorectal cancer (CRC) [49]. Finally, one can validate screening results by identifying evidence that the pathway in question is activated selectively in phenotype-positive tumors, particularly if the pattern of activation in a population of samples is mutually exclusive with the occurrence of other known mechanisms triggering the same phenotype. For example, in a fraction of human melanoma tumors with acquired resistance to RAF or RAF+MEK inhibition, we found evidence of Notch1 activation through elevated expression of the protein and its transcriptional targets. Importantly, tumors with evidence of Notch1-driven resistance did not overlap with those that showed evidence of MAPK reactivation- or PI3K activation-based resistance mechanisms, suggesting that Notch1 may functionally drive resistance in these tumors. [46]. In a similar fashion, we have found evidence of activating mutations in a candidate resistance pathway member, *RAS*, co-occurring with activating mutations in *JAK2* in neoplastic cells from patients with myeloproliferative neoplasms [47]. Tumors bearing this mutation often show intrinsic resistance to JAK inhibition [50, 51], consistent with our results suggesting that concurrent *RAS* mutations may contribute to this resistance. Overall, by using the set of general validation principles described above to provide physiological validation of screening results, we have found that the results of several pathway screens have been confirmed mechanistically and in clinical samples.

## FURTHER APPLICATIONS

To date, we have utilized pathway activating screens to uncover pathways driving resistance to therapy in a broad array of cancer types, and much of the work in our group and others is now leveraging findings from these screens to design more robust therapeutic strategies, some of which are being explored clinically [47, 49]. More broadly, however, this approach has the questions in cancer biology such as the seemingly preferential mutation of certain pathway members, signaling network crosstalk, and the functional impacts of intratumoral heterogeneity. Along with the ability to screen for drug dependencies and mechanism of action of poorly understood therapies [25], it is clear that there exist many applications for this technology outside the field of drug resistance.

Importantly, limitations of the current technology exist. First, while we attempted to include many of the pathways most heavily implicated in tumor biology, the library is nevertheless restricted to pathways of known importance. Thus, it is best viewed as a tool for mapping these pathways to phenotypes of interest rather than as an approach to define new signaling pathways. Moving forward, it will be important to expand the library to include additional pathways such as those controlling epigenetic, metabolic, and transcriptional processes. Also, commonly occurring mutations and alternative pathway nodes may affect the function of a gene and hence, a pathway, differently than the methods of activation or inhibition that are already included in the library. To address these limitations, we have partnered with the National Cancer Institute's Ras Program to expand the current library several times over and make these reagents broadly available to the scientific community. We hope this resource will make further discoveries possible with the ultimate goal of improving our understanding of cancer biology and, hence, therapies for cancer patients.

Functional genomics has proven to be a valuable complement to large-scale sequencing and traditional hypothesis-driven cancer research, particularly as it becomes more clear that a personalized medicine approach will be critical not only for first line therapy, but also for the continued management of patient tumor heterogeneity and evolving resistance. Regardless of the challenges of managing diverse disease types, research has shown that cells remain dependent on a core set of signaling pathways for their continued growth and survival. Harnessing the power of screen-based functional genomics techniques to interrogate the relative importance of these pathways in a multitude of settings will be an important component of the development of effective personalized medicine.

## MATERIALS AND METHODS

### Library construction and validation

To construct the library, cDNA templates for each construct were obtained, barcoded, and cloned into a common expression vector using the Gateway system. Using polymerase chain reaction (PCR), barcodes and relevant Gateway cloning sites were added to each cDNA sequence. The attB1 primers contained the attB1 sequence, a 4-nucleotide (nt) barcode assigned to individual constructs followed by a 14-nt common linker sequence containing a Kozak sequence and ~21 nt of the open reading frame (ORF) of interest. The reverse, attB2 primer contained the attB2 sequence, a C-terminal V5 epitope tag if the cDNA was lacking an epitope tag and the final 21 nt of the ORF (no stop codon) or only the attB2 sequence and the final 24 nt (including the stop codon) if no tag was desired. Where applicable, both tagged and untagged versions of each ORF were functionally validated (Table 1). The resultant PCR fragment was gel purified and transferred to the entry vector, pDONR223, using the BP recombination reaction (Invitrogen). The generated entry clones were sequence verified with the primers, M13-F and M13-R. We ensured the correct sequence of the entire ORF, proper integration of the barcode sequence and the in-frame translation of all elements. If the proper mutations were not present in the original cDNA (as was the case for SMO and LATS2), they were added to the ORF in the entry clone using the QuikChange II XL Site Directed Mutagenesis Kit (Agilent). All mutations were sequence verified. Verified entry clones were submitted to the LR reaction using LR clonase (Invitrogen) to transfer the ORF to a suitable expression vector. All ORFs were transferred to the vector, pcw107-V5, which contains the promoter, PKG, to achieve physiological-like expression levels of the ORF. Expression vectors were fully sequenced with the primers, PGK-F and WPRE-R, and ORF specific internal primers as necessary.

In order to functionally validate the members of the library, lentivirus particles containing the clones were made using a three plasmid system of the expression clone, VSV-G and δVPR, to transfect 293T cells as previously described [23]. Viral particles were titered by limiting dilution in UACC-62 cells. To measure expression of each ORF, immunoblot of the epitope tag, V5, or the encoded protein itself, was performed on whole cell lysates from 293T cells stably and individually expressing each ORF. The pathway activating ability of the constructs was validated using the assays described in Table 1 after puromycin selection of transduced 293T cells. All infections were done by adding a 1:10 to 1:20 dilution of lentivirus particle-containing culture media and 7.5μg/mL polybrene to 293T cells in 6-well plates. Plates were centrifuged at 1200xg for 1 hr at 37°C. After 24 hours, 2μg/mL puromycin was added and cells were incubated for an additional 48 hours before validation assays were performed.

### Primary screens

A pooled lentiviral library was constructed by titering all pathway activating constructs and control constructs individually and then combining them for approximate equal representation. The pooled library was aliquoted and stored at −80°C for use in all primary screens. To screen a particular drug/cell line combination, cells were seeded at 500,000 cells per well in 6-well plates and infected at a multiplicity of infection (MOI) of 0.3 as described above. This MOI predicts that the majority of cells will only receive one construct. After puromycin selection, the surviving cells were lifted and divided into 7 equal populations. One group, representing the *t* = 0 infected pool, was frozen at −80°C. The other six were plated in 6-well plates. Three wells received drug in the range of GI20 to GI80 while the other received diluent only (typically, dimethyl sulfoxide (DMSO)). Drug, vehicle and media were changed every 3 days for 2 to 4 weeks. Cells were split at 1:10 as they became confluent. Samples were then trypsinized and washed and genomic DNA was collected using the Qiagen DNeasy Blood and Tissue Kit.

In order to prepare samples for Illumina Sequencing, we PCR amplified construct barcodes using a common P5 Illumina adapter primer, PGK-Illumina-F, and a unique P7 Illumina barcoded adapter primer, P7- Illumina-RIP-Index-X (where X is a unique numerical identifier). Each screen replicate was paired with a unique P7-reverse primer containing a 6-nt index primer in order to allow sequencing of several samples per lane of Illumina sequencing. Producing a 250-nt fragment, the P5-forward primer is specific for the PGK primer and the P7-reverse primer binds the ATG region of the ORF. These sequences flank the ORF specific barcode. The DNA fragment were purified and size confirmed by 2% agarose gel electrophoresis (Qiagen). Normalized sample pools were submitted to sequencing using band intensities to quantify relative sample amounts. Targeting the region of DNA upstream of the 4-nt ORF barcode, the Illumina Sequencing Primer, ISP, was used to generate a 27-nt Illumina HiSeq read containing the 4-nt barcode, a 17-nt linker sequence and ATG site and the 6-nt index primer. For technical replication, each sample was prepared with two different P7-reverse primers. We found that both the technical and biological replicates yielded comparable data with r^2^ = 0.9. For each unique sequence representing the 4-nt ORF barcode and the 6-nt index primer, the number of reads was counted and the fractional representation in the screen was determined by dividing by the total number of reads of each index primer (i.e. total number of reads for each sample). Fractional representation in each technical and biological replicate was then calculated and averaged. Finally, the fractional representation of each drug treated sample was normalized to the fractional representation of each ORF in the vehicle treated samples. To identify hits, constructs whose representation was enriched in drug treated *versus* vehicle treated samples were identified. The cutoff for hit calling was defined as the presence of at least one activating construct per pathway conferring greater than 50% enrichment above controls across at least two drug concentrations, as constructs scoring at or above this level were found to be reliably validated in secondary, eight-point growth inhibition 50% (GI50 assays).
